# Buffalo Academy of Medicine

**Published:** 1909-07

**Authors:** Harry R. Trick

**Affiliations:** Secretary


### SOCIETY PROCEEDINGS.

### Buffalo Academy of Medicine

*General Meeting, held June 8, 1909.*

Reported by HARRY R. TRICK, M. D., Secretary.

*Report of the Commission on the Municipal Contagious Hospital:*

Your Commission on the Municipal Contagious Hospital herewith presents a summary of work accomplished for the purpose of the establishment of a hospital for Acute Contagious Diseases.

We have at various times enumerated the reasons why such a hospital should be maintained:

1. Because people in fear of quarantine, if sending for a physician allow their children to go unattended; thus a focus of infection may ever be present for the dissemination of the disease.

2. When such cases are thus reported, the Board of Health placards the house; especially for variola, scarlatina, diphtheria, quarantine is maintained; the patient and all living in the same dwelling are isolated.

As at present conducted, quarantine, rigidly enforced without proper facilities for the segregation and treatment of communicable diseases, is a hardship on the following peoples.

1. If a case of infectious disease necessitating placarding occurs where shop and home are in the same building, business is stopped and the source of income is interfered with.

2. If a case occur in a boarding or lodging house, the house is placarded and the business ruined.

3. If a domestic employee becomes infected, the house in which such employee lives is quarantined, the children are not allowed school attendance and the business of members of the family is interfered with.

4. If occurring in the family of wage earners, their means of livelihood is stopped.

5. If occurring in the family of a char woman, the means of livelihood is stopped.

6. If, as so often happens, the disease develops in a tenement, its spread under conditions of crowding and unsanitary environment is great because the enforcement of quarantine is difficult and impossible.

7. Travelers and residents in hotels in the City of Buffalo are at once placed in a dreadful plight; they must leave the hotel within an hour's notice or the hotel is placarded; the law forbids them to hire a public conveyance without first notifying the owner of the conveyance of the purpose for which it is to be used; the law forbids them to leave the city or to go upon the public streets or in public places; they are not allowed to receive visitors.

A summary of the Medical School Examiners' work from September, 1908, to May, 1909, shows that 1881 children were excluded from the schools, and that 8,291 were recommended for treatment; that 601 cultures of throats were taken.

Pupils were excluded from the schools for the following causes: scarlet fever, 165; diphtheria, 44; chicken pox, 151; measles, 13; mumps, 21; pediculosis, 253; tonsillitis, 259; diseases of the eye, 103; diseases of the skin, 229; exposure to contagious diseases, 616; miscellaneous, 87.

Does not this table throw an interesting light upon the creation of the present epidemic of scarlet fever?

The cases of scarlet fever and diphtheria reported from October, 1908, to June, 1909, were as follows:

October, 1908, scarlet fever, 326; diphtheria, 136; November, 1908, scarlet fever, 409; diphtheria, 134; December, 1908, scarlet fever, 329; diphtheria, 87; January, 1909, scarlet fever, 410; diphtheria, 115; February, 1909, scarlet fever, 450; diphtheria, 112; March, 1909, scarlet fever, 440; diphtheria, 79; April, 1909, scarlet fever, 343; diphtheria, 41; May, 1909, 258; diphtheria, 33.

We reiterate that it is the exception rather than the rule that quarantine is strictly followed in these diseases under the present conditions.

On February 21, the Board of Health, by stress of an epidemic, converted an abandoned school house (No. 41) at the corner of Broadway and Spring streets, for use in the care and treatment of cases of scarlet fever. Although this hospital is anything but ideal, the good accomplished is very great, and it is no exaggeration to say that since it was opened the condition, so far as scarlet fever is concerned, has much improved. It has demonstrated that the city under the direction of the Department of Health is competent to conduct such a hospital; that the individual cases have been adequately treated; that those in the immediate household of those infected were enabled to pursue their various vocations unmolested, that children in the household did not lose school time.

It has also been demonstrated that the citizens have faith in such a hospital, for no sooner were the doors opened than the children and adults, the rich and poor, a few—the children of prominent physicians—were admitted for treatment. This proves to your commission that our figures, taken from the London Asylum Boards, statistics of the London Fever Hospitals, that if the city establishes a hospital and manages it intelligently, 80 per cent. of all the infectious diseases reported will seek treatment in such a hospital.

The city has further demonstrated that such a hospital can be administered economically, for the ward cases were treated at about $1.00 per capita per diem. This is an excellent showing and deserves the commendation of the Buffalo Academy of Medicine.

What has been accomplished by your Commission is as follows: Large bodies of organisations were educated into the belief of the necessity of such a hospital. We can give assurance that the interest manifested by the Erie County Medical Society, the Manufacturers' Club, the East Side Business Men's Association, the Esculapian Medical Club, The Civic Federation of Women's Clubs, comprising some 7,000 of the most prominent women in Western New York and including such clubs as the Mothers' Club, the Nurses' Association, the Women's Investigating Club, the Women Physicians' League, the Women Teachers' Association. Then the United Trades and Labor Council, comprising some 30,000, the Cigarmakers' Union No. 2, the Italian-American Business Men's Association, the Equality Club have shown intense interest for the establishment of such a hospital, and these organizations are prepared to coöperate at any time, should they be called upon.

The petition of the Buffalo Academy of Medicine and the Erie County Medical Society, urging the Board of Aldermen to take immediate steps for the establishment and maintenance of a city hospital for infectious and contagious diseases, after public hearing was acted upon favorably by the Board of Aldermen, the Board of Councilmen, His Honor the Mayor, and the Corporation Council was requested to draft a bill as follows: This bill known as Senate Bill 432, Chapter 116, is entitled: An Act authorising the City of Buffalo to construct, equip and maintain a Municipal Hospital or Hospitals for the exclusive care and treatment of persons affected with infectious diseases, except tuberculosis, to acquire lands therefore, and authorising said city to borrow money for such purpose by the issue of bonds, etc. This bill was passed with the approval of the Governor, March 25, 1909.

This, then, gave the city permission to bond itself for the sum of $200,000 to acquire lands and to construct and equip suitable buildings for a Municipal Contagious Hospital for the purpose specified in the title of the act.

The Mayer's Commission (consisting of His Honor the Mayor; Mr. Coppins, President of the Common Council; Mr. Weimar, Chairman of the Board of Aldermen; Dr. Bingham, President of the Board of Councilmen; Dr. Porter, Chairman of the Committee on Sanitary Affairs; Dr. Fronczak, Acting Health Commissioner; Col. Ward, Commissioner of Public Works, to whom was referred the details of this proposed hospital), after deciding upon the necessity for a hospital for acute contagious diseases and after reviewing a number of sites submitted for such a hospital, by unanimous vote, selected a site known as the Best street site (offered by the German Roman Catholic Orphan Asylum for the sum of $50,000) as in their judgment the best as to surroundings and location for such a purpose.

This site is located near Humboldt Park between West Parade avenue and Wohlers street, having 563.5 feet on Dodge street and 486 feet on Best street, being 597.67 feet deep and containing 7 ½ acres of land.

A recommendation that the city purchase this site for the sum of $50,000 was therefore sent to the Board of Aldermen. As soon as the selection of this site was made public, a number of property owners in the vicinity of the site offered the following petition, unfortunately headed by a physician also living in the vicinity of the proposed site:

*To the Honorable, the Common Council of the City of Buffalo:*

We, the undersigned property owners of the vicinity of the Roman Catholic Orphan Asylum, hereby respectfully but vigorously and emphatically object to the proposed erection of a hospital for contagious diseased patients on the tract between Best and Dodge streets, west of Parade Avenue, as such a hospital would be injurious to health and to the value of adjacent property.

We are at a loss to understand why the trustees of the German Roman Catholic Asylum should and would tolerate a hospital of this nature in the immediate vicinity of an asylum which houses hundreds of children of all ages and we, as citizens of one of the most beautiful and respectable portions of the east side hereby oppose any effort to place such hospital at our doors.

Your commission challenges both contentions of this petition as absurd and without the foundation of fact and truth, and as existing as purely fanciful and imaginative due to ignorance or selfishness. Before the Public Hearing held by the Committee of Sanitary Affairs, Dr. Henry R. Hopkins, Dr. Francis E. Fronczak, Acting Commissioner of Health, and the Chairman of this Commission, gave a truthful presentation of facts based upon experience of other cities having Municipal Hospitals, and showed conclusively:

1. That such a hospital is not injurious to the health of those living in the immediate vicinity but that, on the contrary, the zone surrounding such a properly conducted hospital shows fewer contagious diseases reported than outlying zones.

2. That instead of depreciating the value of adjoining property, the land values are appreciably increased. Instead of having a barren and waste piece of land, the landscape gardening which the city will do makes of the place a beauty spot.

Your commission wishes to submit the following letters as bearing upon this important statement:

From Doctor John H. McCullom, Superintendent of the Hospital of the South Department for Contagious Diseases in Boston:

(This is probably as fine a contagious hospital as there exists, and is conducted under the superintendency of Dr. McCullom, an authority on contagious diseases, lecturing upon this subject at Harvard College.)

In reply to your letter, I have to say that in the immediate vicinity of the South Department land in 1893 was assessed at $1.50 per square foot. In 1908, it was assessed at the rate of $2.00 per square foot. The assessed value in Boston is always lower than the actual value.

I send you a diagram showing the houses in the immediate vicinity of the South Department. Northampton Street is about 60 feet wide, and it is the back entrance of the South Department. Since the South Department was opened on Northampton street, quite a number of wooden buildings have been torn down, and very good modern apartment houses have been erected instead.

Doctor Robert J. Wilson, Superintendent of Hospitals of New York City, writes as follows:

Replying to your favor and the question as to the depreciation of land values adjacent to the Kingston Avenue Hospital, I have to say that at the time the Kingston Avenue Hospital property was acquired it was a farm in a low lying swamp, and the property round about it was not very valuable, but there were objections raised by the property owners and the city paid certain damages to the owners of property immediately adjoining that of the hospital grounds. In the last three years, since I have been superintendent of the institution, there have been erected within one hundred and fifty feet of the hospital grounds several tenement houses, and within 1 500 feet of the grounds a large number of dwelling houses.

As I stated in my former letter there has not been any contagious disease among the people of these houses immediately contiguous to this hospital. Of course this is a coincidence, but a very fortunate one, which proves that contagious diseases do not spread from the hospital. The very fact that these modern dwelling houses have been built so near is proof positive in my mind that the property is advancing rather than depreciating in value.

I imagine that the information regarding the acquisition of the Kingston Avenue Hospital could be obtained from the city records of the City of Brooklyn, but conditions are not the same as when this property was acquired. The various physicians in charge of the contagious diseases did not recognise the safety with which contagious disease could be handled in a thickly populated area, if handled as we do now.

I should think that the scarlet fever epidemic in Buffalo this winter must have cost as much as the contagious disease hospital which you contemplate building.

ROBERT J. WILSON,
Superintendent of Hospitals, New York.

The city is pledged to the erection of a hospital for acute communicable diseases. Your commission has been patient and most tolerant of the vexatious delays thus far displayed toward the final accomplishment of what is universally conceded as a pressing necessity.

The present epidemic of scarlet fever will abate, no doubt, with the closure of the schools for the summer vacation, but we predict that it "slumbereth not nor sleepeth," for as soon as the schools are reopened in the fall, contagious disease epidemics, particularly scarlet fever, will continue. Is the will of the people to be dictated by the few? Let the dictum "Buffalo Means Business" be our watchword for the early establishment of a hospital for contagious diseases. Let each member of the profession demonstrate by word and deed our belief that such a hospital is a necessity of the greatest importance for the health and welfare of the citizens of Buffalo.

Let us pray that the Board of Aldermen, the Board of Councilmen, and His Honor, the Mayor, give further proof of their interest by a favorable and speedy action. You do not hesitate to maintain at great expense a Police and Fire Department. These are liberally provided with everything to make their work complete and of value to the public. If these departments are made perfect by liberal appropriation for the protection of our property, why, then, should not that department, whose only object is to protect health and the lives of the whole community, receive as substantial support?

JULIUS ULLMAN,
DEWITT H. SHERMAN,
ROBERT F. SHEEHAN, JR.,
ALLEN A. JONES,
GROVER WENDE.

After reading the report, the following motion was made and carried:

*Resolved,* That the Buffalo Academy of Medicine request the Mayor's Committee to proceed with the greatest possible speed to purchase the site and begin erection of the Municipal Hospital for Contagious Diseases.

